# Implementation of a Competency-Based, Interdisciplinary Pediatric Palliative Care Curriculum Using Content and Format Preferred by Pediatric Residents

**DOI:** 10.3390/children5120156

**Published:** 2018-11-22

**Authors:** Meaghann S. Weaver, Christopher Wichman

**Affiliations:** 1 Division of Pediatric Palliative Care, Children’s Hospital and Medical Center, Omaha, NE 68114, USA; 2 Division of Biostatistics, University of Nebraska Medical Center, Omaha, NE 68198, USA; christopher.wichman@unmc.edu

**Keywords:** pediatric palliative care, resident education, social cognitive theory

## Abstract

Palliative care competencies at the pediatric resident training level expand learned knowledge into behavior. The objective of this study was to investigate mode of palliative care education delivery preferred by pediatric residents and to report on participatory approach to resident palliative care curriculum design. A one-hour monthly palliative care curriculum was designed and implemented in a participatory manner with 20 pediatric residents at a free-standing Midwestern children’s hospital. Outcome measures included pediatric residents’ personal attitude and perceived training environment receptivity before and after implementation of a palliative care competency-based curriculum. An 18-item survey utilizing Social Cognitive Theory Constructs was administered at baseline and after palliative care curriculum implementation (2017–2018 curricular year). Pediatric residents prioritized real case discussions in group format (16/20) over other learning formats. Topics of highest interest at baseline were: discussing prognosis and delivering bad news (weighted average 12.9), pain control (12.3), goals of care to include code status (11.1), and integrative therapies (10.7). Summary of ordinal responses revealed improvement in self-assessment of personal attitude toward palliative care and training environment receptivity to palliative care domains after year-long curriculum implementation. Curricular approach which is attentive to pediatric residents’ preferred learning format and self-assessment of their behaviors within their care setting environment may be beneficial in competency-based primary palliative training.

## 1. Introduction

Curricular models have recently been developed for palliative care competencies in medical school and residency programs [1,2]. Formal palliative care teaching positively impacts medical residents’ knowledge and comfort level with palliative care [3]. A prior study exploring six hours of palliative care education revealed statistically significant improvement in pediatric resident learning and confidence after curricular implementation [4]. A competency-based palliative care education emphasizes not only a knowledge base, but an actual behavior and a perception of feasibility within the care setting environment so that knowledge becomes an action [5]. Competency-based curricula ideally reach bedside impact, as a mandatory core pain management palliative care curriculum for surgical residents was found to improve patient perception of pain control [6].

Behavioral theory has been under-utilized in curricular design. Social Cognitive Theory (SCT) explains how people acquire knowledge and maintain certain behavioral patterns (competencies) as a result of that acquired knowledge [7]. Social Cognitive Theory recognizes three main factors which influence the way that knowledge becomes action: affective realities of each learner, social and physical environments, and behavioral factors [8]. The main principles of SCT which apply to pediatric resident palliative care competencies include the following:

- Self-efficacy: the pediatric resident feels confident introducing principles of primary palliative care.
- Behavioral capability: the pediatric resident believes he/she can obtain the knowledge and skill to integrative palliative care principles as part of his/her training.
- Intentions (proximal goals): the pediatric resident intends to incorporate palliative care principles into patient care.
- Situation (perceived environment): the pediatric resident perceives opportunities for integrating palliative care into patient care.
- Social support: the training setting encourages the pediatric resident to consider palliative care principles in patient care and fosters a learner applying palliative care principles during residency years.
- Outcome expectations: the pediatric resident believes exposure to palliative care principles improves patient care (professional development) and enhances sense of resiliency (personal wellness).
- Expectancies: the learning community places value on the potential outcome of palliative care principles for patient care.

The purpose of this research project was to partner with pediatric residents to prioritize palliative care learning topics and training format. The goal was to then apply principles from SCT to develop a monthly palliative care curriculum (Pal Care Power Hour) with pediatric residents and to measure impact of this curriculum using SCT constructs. This study of pediatric resident palliative care curriculum outcomes measured residents’ self-assessed attitude about palliative care and their perceptions of environmental receptivity to palliative care.

## 2. Materials and Methods

A group of 20 residents met with the palliative care provider prior to team development to determine content and format of the Pal Care Power Hour year-long curriculum in two separate planning sessions prior to curricular roll-out. The residents were asked to prioritize and strategize curricular development according to primary palliative care competencies [9]. Twelve educational sessions then occurred on the third Thursday of each month for one hour with lunch provided. The curriculum consisted of distribution of three journal articles relevant to each month’s educational topic one week prior to the in-person learning lunch. Each monthly Pal Care Power Hour in-person session consisted of: a real life palliative care actionable case example (behavioral emphasis), topical palliative care content learning relevant to the case (cognitive emphasis), and group discussion for application in local care setting (environmental emphasis). The curriculum topical list is provided as Appendix A.

A two-part instrument comprised of environmental and behavioral SCT content items was distributed to pediatric residents between July 2017–June 2018. Survey questions were designed according to the Tailored Method of Survey Design [10]. The survey content included one question on training year; one question on preferred learning format and another on preferred learning topics; and the remainder of questions were regarding SCT attitude or environmental constructs. This instrument has been validated as a SCT theory tool for nutritional educational assessment but not specifically for palliative care educational assessment. In nutritional education studies, Cronbach’s α for the survey instrument were >0.7 [11]. The Pal Care Power Hour survey was independently reviewed, piloted, revised, and re-piloted by three internal medicine residents as a test group for survey design prior to administration to pediatric residents on SurveyMonkey (San Mateo, CA, USA). A link to the questionnaire was sent in an introductory email inviting pediatric resident participation in the survey with two reminder emails sent in two week intervals. The survey instrument is available in full-text as Supplementary Materials Questionnaire S1.

Data was downloaded from SurveyMonkey into Microsoft Excel (Microsoft Office, Redmond, WA, USA) and Statistical Package for the Social Sciences (SPSS) version 21 (IBM, Armonk, NY, USA) for analyses. The analyses were primarily grounded in tallying counts and proportion calculations. The study team utilized descriptive statistics and counts for categorical variable responses. Ordinal responses were summarized by count and percentage for the pre- and post- responses separately per statement. Due to the anonymous nature of the surveys, and the resultant loss of pairing, statistical tests were not performed.

The Office of Human Subjects Research Protections determined that the survey format and content qualified as exempt from full Institutional Review Board review due to anonymity of responses and survey inclusion in a programmatic quality improvement initiative.

## 3. Results

A total of 20 pediatric residents (nine interns, five second years, and six third years) completed the pre- and post-assessment. Upfront response rate was 65% overall. For those twenty residents who completed a pre-intervention survey, the response rate for post-intervention survey was 100%.

Preferred educational format for the Pal Care Power Hour was noted to be real case discussions in group format (16/20) over lectures (1/20), journal discussion (1/20), panel speakers (1/20), and simulated learning (1/20). Surveys revealed the prioritized topics ranked as: discussing prognosis and delivering bad news (weighted average 12.9), pain control (12.3), goals of care to include code status (11.1), and integrative therapies (10.7). This prioritization of curricular topics resulted in participatory development of the teaching content (Appendix A). 

**Figure 1:**
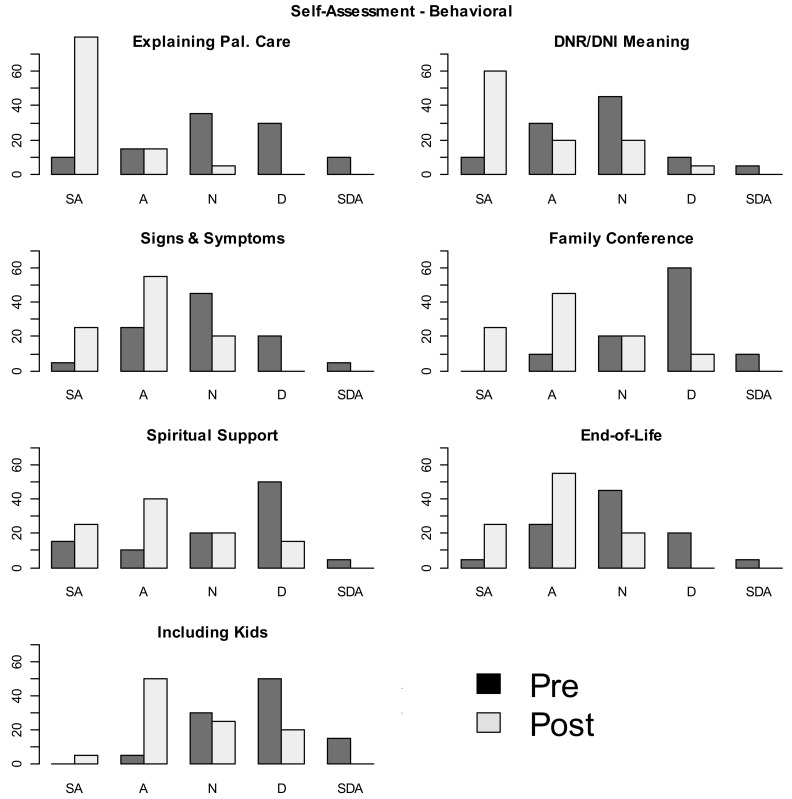
Pediatric resident personal comfort with palliative care competencies pre- and post-curriculum implementation. DNR = Do Not Resuscitate, DNI = Do Not Intubate. Note that the Y-Axis refers to percentage of responses.

**Figure 2:**
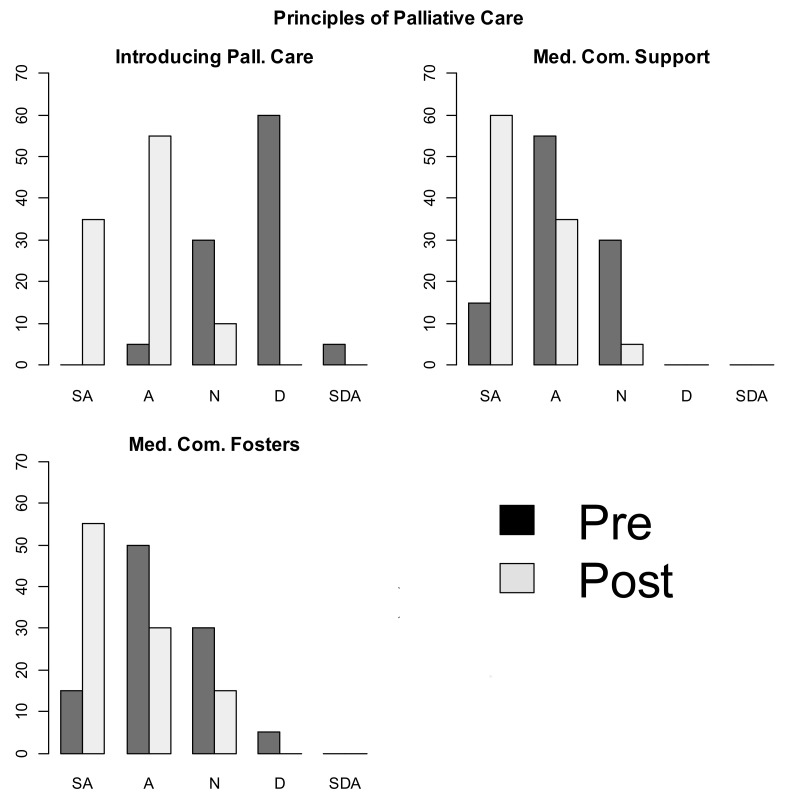
Perceived environmental receptivity to palliative care principles pre- and post-curriculum implementation. Abbreviation: Med. Com. Support = Medical Community Supports Palliative Care Principles; Med. Com. Fosters = Medical Community Fosters Palliative Care Principle Integration. Note that the Y-Axis refers to percentage of responses.

Pre-post intervention side-by-side boxplots depicting shift in personal attitude and perception of environmental attitude are presented as Table 1. There was a visible shift in personal learner attitudes about palliative care principles pre- and post- curricular implementation (Figure 1). There was also a visible shift in how pediatric residents perceived their training environment’s receptivity to pediatric palliative care principles (Figure 2) from pre- to post- lecture implementation. The shift in SCT principles did not correlate with pediatric resident training level or gender.

## 4. Discussion

By developing a Pal Care Power Hour curriculum in a participatory manner with resident input on learning format and topical content, the research fostered a competency-based training experience. Based on overwhelming resident preference for real life case discussions over lecture format, the curricular design was interdisciplinary case review with real life application. The interdisciplinary nature of the curriculum included collaborative inclusion of colleagues as co-teachers with the palliative care providers. Examples of interdisciplinary partnership in the teaching include wound care nurses providing hands-on demonstrations of skin procedures and products as part of the dermatology lecture; bereaved parents and grief counselors serving as panel experts during the bereavement education session; and hands-on massage therapy and tai chi sessions from integrative therapists as part of resiliency teaching (Appendix A).

Limitations of this study included single-site investigation, small sample size, lack of pairing of surveys, and use of a scale not specifically validated for palliative care educational content. There were possible unexplored confounders such as not controlling for whether any of the residents participated in a palliative care clinical elective during the year. Strengths of this study included use of behavioral theory, participatory format in curricular design, and exploring palliative care competencies in a context beyond knowledge base.

Newly graduating doctors and incoming pediatric residents depict feeling unprepared to manage patients with palliative care and terminal care needs [12,13]. While there has been significant progress in the amount and quality of palliative care education offered in medical schools, resident physicians express a need for ongoing palliative care education implemented longitudinally in their training [14]. Problem-based learning with interdisciplinary educators has been endorsed as a form of palliative care education for pediatric trainees [15]. In a prior study, pediatric residents shared the need for improved training in discussing prognosis, delivering bad news, and managing physical pain as essential areas of foundational knowledge [16]. Communication and symptom management were identified by pediatric residents as key palliative care knowledge needs worthy of special teaching both in lecture format and at the bedside [17]. Prior research revealed that a structure pediatric palliative care curriculum for practicing pediatric clinicians increased pediatricians’ self-efficacy, knowledge, and emotional comfort with palliative care topics [18]. The pediatric residency years are a prime time to foster integration of palliative care curricula as part of professional and personal formation for residents.

Effective primary palliative care curricular development for pediatric residents recognizes human capacities for knowledge while also acknowledging the interaction between learners, their beliefs, and their social environments for actual learning. By using SCT attitude and environment constructs, this research study explored not only palliative care knowledge base but also the learner’s sense of training environment expectancies and receptivity. For a pediatric trainee to meaningfully apply a palliative care competency at the bedside, the trainee ideally has developed a sense of confidence, capability, supportive environment, positive intention, and meaningful expectation for behavioral implementation. Pediatric residents were able to actively co-design a palliative care curriculum which resulted in a positive impact to their own comfort with pediatric palliative care principles and their sense of environmental support for implementation of palliative care principles.

## Figures and Tables

**Table 1:** Environment and attitude pre- and post-palliative care year-long curriculum.

Domain	Principles of Palliative Care	Timing	Count (%)				
			SA	A	N	DA	SDA
Attitude	I feel confident introducing principles of palliative care	Pre	0 (0)	1 (5)	6 (30)	12 (60)	1 (5)
		Post	7 (35)	11 (55)	2 (10)	0 (0)	0 (0)
Attitude	I believe I can obtain the knowledge and skills to integrate palliative care principles	Pre	4 (20)	12 (60)	3 (15)	1 (5)	0 (0)
		Post	8 (40)	10 (50)	2 (10)	0 (0)	0 (0)
Attitude	In the next 3 months, I intend to incorporate palliative care principles in my care for patients and families	Pre	4 (20)	13 (65)	3 (15)	0 (0)	0 (0)
		Post	9 (45)	9 (45)	2 (10)	0 (0)	0 (0)
Attitude	I believe exposure to palliative care principles improves my care of patients and families (professional development)	Pre	14 (70)	4 (20)	2 (10)	0 (0)	0 (0)
		Post	15 (75)	4 (20)	1 (5)	0 (0)	0 (0)
Attitude	I believe exposure to palliative care principles enhances my own sense of resiliency (personal wellness)	Pre	10 (50)	9 (45)	1 (5)	0 (0)	0 (0)
		Post	16 (80)	4 (20)	0 (0)	0 (0)	0 (0)
Attitude	I place value on the potential outcome of palliative care principles for patient care	Pre	9 (45)	10 (50)	1 (5)	0 (0)	0 (0)
		Post	15 (75)	5 (25)	0 (0)	0 (0)	0 (0)
Environment	The medical community supports me in my learning about palliative care	Pre	3 (15)	11 (55)	6 (30)	0 (0)	0 (0)
		Post	12 (60)	7 (35)	1 (5)	0 (0)	0 (0)
Environment	The medical community fosters my applying palliative care principles	Pre	3 (15)	10 (50)	6 (30)	1 (5)	0 (0)
		Post	11 (55)	6 (30)	3 (15)	0 (0)	0 (0)
Environment	I perceive opportunities for integrating palliative care for children and families	Pre	5 (25)	12 (60)	2 (10)	1 (5)	0 (0)
		Post	11 (55)	8 (40)	0 (0)	1 (5)	0 (0)
Environment	This hospital encourages me to consider palliative care principles in my care of patients and families	Pre	3 (15)	9 (45)	8 (40)	0 (0)	0 (0)
		Post	10 (50)	8 (40)	2 (10)	0 (0)	0 (0)

SA = strongly agree; A = agree, N = neutral, DA = disagree; SDA = strongly disagree.

## Supplementary Materials

The following are available online at http://www.mdpi.com/2227-9067/5/12/156/s1, Questionnaire S1: Lecture topics of interest.

## Appendix A. Pediatric Pal Care Power Hour Learning Series Content

1. Delivering hard news with children and families (discussing pediatric prognosis).
2. Discussing and documenting goals of care to include child’s code status.
3. Integrative therapies and staff self-care/resilience (mindfulness, guided imagery, massage therapy, tai chi, reflexology, Healing Touch and Reiki principles); co-taught with integrative therapists.
4. Pediatric complex pain control with pharmaceutical focus (adjuvant therapies for pain, opiate selection and rotation); co-taught with acute pain service and anesthesiology team.
5. Treatment of respiratory symptoms at a child’s end of life (secretions, dyspnea); co-taught with pediatric respiratory therapy team.
6. Treatment of pediatric gastrointestinal symptoms at end of life (constipation, malignant bowel obstruction, anorexia, cachexia); co-taught with gastrointestinal nurse practitioner.
7. Treatment of pediatric neurologic symptoms at end of life (agitation, delirium, anxiety); co-taught with pediatric psychiatry team.
8. Treatment of pediatric dermatologic symptoms at end of life (intractable pruritis, wound care, skin breakdown); co-taught with wound care team.
9. Recognizing and preparing for a child’s imminent death (anticipatory guidance on physical changes).
10. Existential and spiritual distress in children and families with a focus on cultural respect; co-taught with spiritual ministry team.
11. Pediatric home hospice care; co-taught with community hospice representatives.
12. Bereavement care for siblings, grandparents, and child’s peers; co-taught with bereaved parent panel and with grief counselors.
