# Supplementary material for: Implementation of a Competency-Based, Interdisciplinary Pediatric Palliative Care Curriculum Using Content and Format Preferred by Pediatric Residents
Source: Children (Basel). 2018 Nov 22;5(12):156. doi: 10.3390/children5120156 (PMC6306952; doi:10.3390/children5120156)
Supplement: Supplementary file 1 [file children-05-00156-s001.pdf]

Lecture Topics of Interest

Hi, Residents --- We are so excited to introduce a palliative care lecture series on the third Wednesday of each month. . . starting in July! Please kindly complete the attached anonymous survey to ensure that the monthly pal care power sessions are catered to your interests and comfort level. The survey is expected to take <10 minutes total. There are prizes for five survey respondents (IF you chose to enter your name --- optional).

1. My current level of training. . .

[ ]

2. Please rank the top 5 topics you would be interested in for palliative care learning sessions (1= most interested)

- [ ] Delivering bad news and discussing prognosis
- [ ] Discussing and documenting code status
- [ ] Integrative therapies (mindfulness, guided imagery, massage therapy, tai chi, reflexology, Healing Touch, and Reiki principles)
- [ ] Pain control - pharmaceutical (adjuvant therapies for pain, opiate selection and rotation)
- [ ] Treatment of Respiratory symptoms at end of life (secretions, dyspnea)
- [ ] Treatment of GI symptoms at end of life (constipation, malignant bowel obstruction, anorexia, cachexia)
- [ ] Treatment of Neurological symptoms at end of life (agitation, delirium, anxiety)
- [ ] Treatment of Dermatologic symptoms at end of life (intractable pruritus, wound care, skin breakdown)
- [ ] Recognizing and preparing for imminent death (anticipatory guidance on physical changes)
- [ ] Existential and spiritual distress - with a focus on cultural respect
- [ ] Home hospice care - what does home hospice do and what does this mean for children?
- [ ] Staff resilience and self-care
- [ ] Forgotten family members - the literature base on sibling support and grandparent needs
- [ ] Bereavement care

3. What other palliative care type topics would be helpful/important?

Learning Style Preference

4. Rank your top 5 preferred methods of learning (1 = most preferred)

- [ ] Lecture format
- [ ] Journal article discussion
- [ ] Discussing and problem solving with real life examples
- [ ] Conversation as a resident group
- [ ] Case studies in small groups
- [ ] Simulation using actors
- [ ] Interdisciplinary guest speakers representing various fields (chaplains, grief counselors, social workers, family members, etc.)

Current level of comfort

5. For the next section, "principles of palliative care" are defined as: "principles which focus on providing relief from the symptoms and stress of a serious illness with a goal to improve quality of life for both the patient and the family."

	Strongly Agree	Agree	Neutral	Disagree	Strongly Disagree
I feel confident introducing principles of palliative care	○	○	○	○	○
I believe I can obtain the knowledge and skills to integrate palliative care principles	○	○	○	○	○
In the next 3 months, I intend to incorporate palliative care principles in my care for patients and families	○	○	○	○	○
I perceive opportunities for integrating palliative care for children and families	○	○	○	○	○
This hospital encourages me to consider palliative care principles in my care of patients and families	○	○	○	○	○
The medical community supports me in my learning about palliative care	○	○	○	○	○
The medical community fosters my applying palliative care principles	○	○	○	○	○
I believe exposure to palliative care principles improves my care of patients and families (professional development)	○	○	○	○	○
I believe exposure to palliative care principles enhances my own sense of resiliency (personal wellness)	○	○	○	○	○

	Strongly Agree	Agree	Neutral	Disagree	Strongly Disagree
I place value on the potential outcome of palliative care principles for patient care	○	○	○	○	○

Self Assessment

6. Self-Assessment

	Strongly Agree	Agree	Neutral	Disagree	Strongly Disagree
I am confident explaining to a family what palliative care is and their role	○	○	○	○	○
I am able to describe the difference between palliative care and hospice	○	○	○	○	○
I am knowledgable on how and when a goals of care conversation is needed	○	○	○	○	○
I am comfortable explaining to a family what DNR/DNI means and discussing code status	○	○	○	○	○
I have an ability to recognize the signs and symptoms indicating that a patient's death is imminent	○	○	○	○	○
I am comfortable providing adequate pain control for pediatric patients imminent at end of life	○	○	○	○	○
I am comfortable controlling other symptoms (dyspnea, secretions, anxiety, agitation, skin changes) during a child's dying process	○	○	○	○	○
I have access to resources regarding non-pharmacological methods of pain control at the hospital (integrative therapies)	○	○	○	○	○
I am aware of protocoled approaches for sharing "bad news"	○	○	○	○	○

	Strongly Agree	Agree	Neutral	Disagree	Strongly Disagree
I feel capable conducting a family conference to discuss the new diagnosis of a life-threatening illness	○	○	○	○	○
I am aware that there is a broad range of cultural and religious attitudes and practices regarding death	○	○	○	○	○
I am comfortable providing spiritual support for dying children and their families	○	○	○	○	○
I am capable of participating in end-of-life discussion	○	○	○	○	○
I am confident including children in discussion about preparing for death	○	○	○	○	○
I am aware of the unique needs of siblings and grandparents of patients with life-limiting illnesses	○	○	○	○	○
I am knowledgable about pronouncing when a child is dead	○	○	○	○	○
I am informed about supportive approaches to communicating with a bereaved family after their child has died	○	○	○	○	○
I believe that personal resiliency and self-care are meaningful and important	○	○	○	○	○

Optional

7. You can optionally enter your email address to be added to a drawing list for palliative care textbooks (total of 5 prize winners). This is optional. Thank you so very, very much for the time and energy invested in completing this survey! We hope you enjoy the upcoming Wed lecture series each month. Your responses will guide our developing the palliative care curriculum for residents.
